# University of Maryland Dental Department

**Published:** 1888-11

**Authors:** G. F. S. Wright

**Affiliations:** President of S. C. State Dental Examining Board.


### Editorial, Etc.

University of Maryland Dental Department.—*Another Letter from Dr. G. F. S. Wright, President of South Carolina State Dental Examining Board.*—

"Georgetown, S. C., Nov. 15th, 1888.

*Professor F. J. S. Gorgas, Dean of Dental Department University of Maryland :*

*Dear Doctor.*—The Committee of which Dr. Levy writes, whose report he says I signed. held no meeting when I was present, and the final action was as I stated.

The roll was called, and as the Secretary called the name of an institution, the Presiding Officer, Dr. Waters, would ask if there was any objection, and if there was nothing said he would announce : "*No objection.*" I kept a score of the roll, and no one objected to the University of Maryland Dental Department. I have written this to Dr. Waters, who has written to me and stated his impression of the action. Dr. Levy says the proceedings will be published in the *Ind.* I am not in possession of any information except rumors from the Association of Dental Faculties ; of these the impression made was not unfavorable to the School of which you are the Dean.

That the Dental Department of the University of Maryland was objected to is a mistake, it was not. No School in Baltimore was, but a Washington School did have objection made and was turned down.

I am very respectfully,

G. F. S. Wright, D. D. S.

President of S. C. State Dental Examining Board."

